# Binge, Purge, and Wander: Navigating Bulimia and Attention Deficit Hyperactivity Disorder (ADHD)

**DOI:** 10.7759/cureus.69076

**Published:** 2024-09-10

**Authors:** Daniela Jeremias, Leonor Santana, Francisca Braga, Bárbara Mesquita, Catarina Santos

**Affiliations:** 1 Psychiatry Department, Hospital de Egas Moniz - Unidade Local de Saúde de Lisboa Ocidental, Lisbon, PRT; 2 Psychiatry Department, Hospital de Cascais, Lisbon, PRT

**Keywords:** attention-deficit hyperactivity disorder, bulimia nervosa, lisdexamfetamine, mental health comorbidities, psychostimulants

## Abstract

A 32-year-old woman with no prior medical conditions, but a history of anorexia nervosa (AN) diagnosed in adolescence, was referred for evaluation by an eating disorders (ED) specialist due to worsening bingeing and purging behaviors. Her clinical presentation was characterized by a body mass index (BMI) approaching the underweight range and frequent bingeing and purging episodes occurring twice daily, consistent with a diagnosis of BN. No other medical or psychiatric comorbidities were diagnosed, including mood, anxiety, and substance use disorders, which are often comorbid with ED. However, the patient reported significant difficulties in managing both personal and professional activities, attributing these challenges to impaired concentration. She had difficulty staying focused on tasks and was easily distracted by unrelated thoughts or stimuli. As a result, she often procrastinated on household and self-care tasks. She also reported problems with time management, frequently arriving late to work, struggling to complete assignments on time, and underestimating how long tasks would take.

Initial treatment with fluoxetine and cognitive behavioral therapy (CBT) yielded no substantial improvement. Given the presence of symptoms suggestive of attention deficit hyperactivity disorder (ADHD), a one-month trial of lisdexamfetamine (LDX) was initiated. This intervention resulted in a marked reduction in bingeing and purging episodes and a notable improvement in the patient’s concentration, thereby enhancing her overall quality of life.

The importance of ADHD screening is underscored, particularly for high-functioning adult women who may not present typical symptoms. In this case, a thorough clinical assessment and detailed anamnesis raised suspicion of previously unrecognized ADHD, that may have been present since childhood.

Although the literature on the comorbidity of ADHD and BN is limited, this case highlights a potential link between the two conditions. The significant improvement observed following the introduction of psychostimulants supports the hypothesis that untreated ADHD may contribute to the exacerbation of BN symptoms. Further research is essential to clarify the underlying mechanisms and establish a solid scientific basis for future clinical interventions and therapeutic strategies.

## Introduction

Approximately 1-5% of adults worldwide suffer from attention deficit hyperactivity disorder (ADHD) while about 1% of women are affected by bulimia nervosa (BN) [1,2]. Emerging research supports an overlap between ADHD and BN, pointing out a significant important comorbidity that warrants further investigation. The prevalence of binge/purge behaviors is notably higher in individuals with ADHD (1.9%) compared to the general population (0.4%) [3]. This increased prevalence may be attributed to common risk factors and symptoms, such as impulsivity and difficulties with self-regulation, common to both conditions [3,4].

In standard clinical practice, fluoxetine is the only medication approved in Canada, the United States, and Europe for treating BN, alongside psychological interventions such as cognitive behavioral therapy (CBT) [5].

Despite recognizing this overlap, treatment options for BN, particularly when complicated by ADHD, remain limited. The evidence on how effective fluoxetine is for treating BN, especially when ADHD is also present, is inconsistent. Many patients do not achieve satisfactory outcomes [3,6]. Preliminary studies and reports suggest that stimulants such as lisdexamfetamine (LDX) may reduce binge/purge behaviors in BN, regardless of comorbid ADHD. LDX has emerged as a potential adjunctive therapy for BN [3,6].

We report a 32-year-old woman with BN, whose initial treatment with fluoxetine, topiramate, and CBT did not yield significant improvement. However, the addition of LDX, a psychostimulant used for ADHD, resulted in a substantial reduction in binge/purge episodes. Through this case report, we aim to underline the importance of considering ADHD in patients with BN and to illustrate the potential benefits of psychostimulant treatment for managing comorbid conditions.

## Case presentation

We present the case of a 32-year-old woman with a prior history of eating disorders (ED). She was referred by her general practitioner (GP) to an ED psychiatry appointment due to the worsening of her bingeing and purging, with a suspicion of BN. At our first appointment, her body mass index (BMI) was 18,61 kg/m², and she was engaging in bingeing and purging twice a day. She was already on fluoxetine 20 mg per day without improvement. She viewed her behavior as a way to soothe herself in response to stressful events at work but appeared motivated to improve and undergo outpatient treatment. 

During her infancy, she had a close relationship with her mother, and her grandmother also played an important role in her life. Her disordered eating patterns began at the age of 13, when she moved to a different city with her mother and stepfather, with whom she had a difficult relationship. Her father was never present, so she never met him. Currently, she solely maintains a close relationship with her mother, despite living in different countries.

Her disordered eating patterns started at the age of 13 when she moved to a different city. At that time, she began eating as little as possible and lost a significant amount of weight over a year. She began attending child psychiatry outpatient appointments with a BMI of 14.89 kg/m², which led to a diagnosis of AN restricting type. She recalls being prescribed olanzapine, though she does not remember the dosage and believed it was prescribed due to her distress related to mealtimes. She was also referred for nutritional appointments.

At age 14, she received inpatient treatment and was discharged after three months with a BMI of 17.73 kg/m². She recalls continuing the same medication at a higher dosage. The treatment primarily focused on addressing her dietary needs to stabilize her nutritional status and establish long-term nutritional goals. A few months after discharge, her maternal grandmother, with whom she was very close, passed away. In her grief, she restarted restricting her diet and lost a significant amount of weight, reaching a BMI of 13.82 kg/m². She was again readmitted to the hospital and described that "I couldn't feel emotions...I wasn't exactly sad...but restricting food helped me grieve my grandmother". At admission, while alexithymia might have been present, there were no other criteria for a mood disorder. She was discharged two months later with a BMI of 16.27 kg/m² in her words "feeling sad and very irritable at that time". The patient recalls being prescribed an antidepressant and olanzapine but does not remember the name of the antidepressant, the dosage of the olanzapine, or the duration of the treatment.

She continued her treatment as an outpatient with periods of improvement and relapse. The patient's history describes depressive symptoms following the sudden death of her grandmother, her subsequent hospital readmission, and the resulting weight gain. Apart from that, no other prevalent comorbidities, such as anxiety disorders or substance use disorders, were found. Additionally, obsessive-compulsive disorder (OCD), post-traumatic stress disorder (PTSD), and autism spectrum disorder (ASD) were also excluded. At age 17, two years after her last hospitalization, she began bingeing and purging daily with an average BMI of 16 kg/m². Although she never overexercised, she started taking laxatives and over-the-counter weight loss pills. At this point, her diagnosis changed to AN binge-purge type.

For career reasons, she moved to different countries twice during her twenties. She stopped attending her psychiatry appointments, maintaining a BMI between 16 and 17 kg/m². She started to accept her disease as part of her personality (“I have been like this for so many years that it almost feels as if the disease is part of who I am. But I have moments when I feel well and barely binge and purge, especially when I’m traveling, going from one place to another for months. But when I’m at work sitting all day, it all comes back”).

As mentioned earlier, at our first appointment, her BMI was 18.6 kg/m², and she was bingeing and purging twice daily. Despite being on 20 mg of fluoxetine daily, her condition had not improved. Given that the patient has a healthy BMI, albeit on the lower end of the healthy range, the diagnosis of AN binge-purge type was excluded, and the diagnosis of BN was considered. Additionally, common features observed in individuals with AN such as elevated cholesterol levels; menstrual cycle abnormalities; dry and flaky skin; lanugo; cold sensitivity, and yellowing of the skin were absent. During the physical examination, and despite frequent exposure to stomach acid from repeated vomiting, clinical signs of BN such as swollen cheeks or jawline; bilateral parotid gland enlargement; tooth decay or enamel erosion; Russell's sign, and electrolyte imbalances were also absent.

After beginning to follow this patient, her fluoxetine intake was gradually increased to 60 mg per day, and topiramate was introduced gradually to 50 mg twice a day, without improvement. Regarding her bingeing and purging episodes, she reported having no food preferences and often ordered food through an app during a binge episode, as she reportedly only kept healthy food at home. During appointments, she had trouble staying still, fidgeting, and moving around in her chair. Upon further questioning, she reported difficulty focusing on simple tasks, such as cleaning the house, managing her finances, meeting her friends on time, and focusing long enough to paint, read, or watch movies, some of her favorite hobbies. She described her lifestyle as “chaotic and without schedules, except for work”. These complaints and her mannerisms prompted the diagnosis of comorbid ADHD. She also described herself as an easily distracted child but never had poor grades or issues with teachers or authority. To maintain her academic performance, she frequently paced around her room while reading her notes to better memorize because it would be much harder sitting still at a desk.

No other medical or psychiatric comorbidities were diagnosed and a trial with LDX 30 mg per day was initiated. At her next appointment one month later, she reported improvement within two days. Not only did her ADHD symptoms improve, allowing her to better focus and perform tasks at home and work, but the frequency of her binging and purging episodes also decreased to twice a week.

## Discussion

As mentioned in the introduction, recent literature suggests an overlap between ADHD and eating disorders, particularly BN.

However, ADHD diagnoses often go overlooked in both young and adult females, especially those with higher functioning who tend to exhibit inattentive symptoms. Unlike men, these women often internalize and conceal their symptoms, despite experiencing daily impairment [1,2]. Both girls and women with ADHD may express their distress through disordered eating [2,7].

LDX shows promise as a treatment for BN due to its unique neurobiological mechanism, although its effects beyond appetite suppression are not yet fully understood. Preliminary studies have hypothesized it may involve dysfunctional dopamine and noradrenaline systems, both altered by stimulants [6-8]. While case reports of LDX for BN are encouraging, some experts raise concerns about the appetite suppression side effect and the risk of intentional misuse for weight loss, particularly in patients without ADHD [3,6].

LDX, a psychostimulant approved for ADHD, has recently been approved for treating binge eating disorder (BED) in adults [7]. However, rigorous studies evaluating stimulant treatment for BN are still lacking. The long-term safety of LDX for BN is unknown, though close follow-up and monitoring of patients may be necessary [8-10].

As illustrated by our case, a detailed anamnesis was crucial for identifying previously unsuspected ADHD-like symptoms, possibly present since childhood. Treatment with LDX significantly reduced the frequency of binge/purge episodes and improved her ADHD symptoms. By addressing the underlying condition, her impulsivity and distress levels decreased, targeting the core of her bingeing/purging behaviors. Screening for ADHD in patients with loss of control over bingeing/purging should be required [1,2]. Non-pharmacological ADHD-oriented interventions might also be worth considering [10].

## Conclusions

A late diagnosis of ADHD is not uncommon in the presence of an ED. There is also a higher risk of delayed diagnosis and subsequent mistreatment of ADHD among adult women, who have a greater prevalence of psychiatric comorbidities such as BN. However, treating ADHD offers the potential advantage of addressing both conditions simultaneously, thereby enhancing overall outcomes. Future studies should address whether patients with this comorbidity have a different prognosis, course of illness, and treatment response when compared to patients with either disorder alone.
